# Cellular specificity of lactate metabolism and a novel lactate-related gene pair index for frontline treatment in clear cell renal cell carcinoma

**DOI:** 10.3389/fonc.2023.1253783

**Published:** 2023-09-19

**Authors:** Xiangsheng Li, Guangsheng Du, Liqi Li, Ke Peng

**Affiliations:** Department of General Surgery, Xinqiao Hospital, Army Medical University, Chongqing, China

**Keywords:** lactate metabolism, single cell analysis, gene pair algorithm, mTOR-targeted therapy, immune checkpoint blockade

## Abstract

**Background:**

Although lactate metabolism-related genes (LMRGs) have attracted attention for their effects on cancer immunity, little is known about their function in clear cell renal cell carcinoma (ccRCC). The aim of this study was to examine the cellular specificity of lactate metabolism and how it affected the first-line treatment outcomes in ccRCC.

**Methods:**

GSE159115 was used to examine the features of lactate metabolism at the single-cell level. Utilizing the transcriptome, methylation profile, and genomic data from TCGA-KIRC, a multi-omics study of LMRG expression characteristics was performed. A prognostic index based on a gene-pair algorithm was created to assess how LMRGs affected patients’ clinical outcomes. To simulate the relationship between the prognostic index and the frontline treatment, pRRophetic and Subclass Mapping were used. E-MTAB-1980, E-MTAB-3267, Checkmate, and Javelin-101 were used for external validation.

**Results:**

The variable expression of some LMRGs in ccRCC can be linked to variations in DNA copy number or promoter methylation levels. Lactate metabolism was active in tumor cells and vSMCs, and LDHA, MCT1, and MCT4 were substantially expressed in tumor cells, according to single-cell analysis. The high-risk patients would benefit from immune checkpoint blockade monotherapy (ICB) and ICB plus tyrosine kinase inhibitors (TKI) therapy, whereas the low-risk individuals responded to mTOR-targeted therapy.

**Conclusions:**

At the single-cell level, our investigation demonstrated the cellular specificity of lactate metabolism in ccRCC. We proposed that the lactate-related gene pair index might be utilized to identify frontline therapy responders in ccRCC patients as well as predict prognosis.

## Introduction

Metabolic disorders are widely involved in the occurrence and development of various diseases (1, 2), and recent evidence has accumulated that metabolic disorders in cancer cells are not only a hallmark of cancer, but may also be the fundamental cause of tumors. In 1923, Otto Warburg observed that tumor cells tend to take up large amounts of glucose and produce excess lactic acid through anaerobic glycolysis. This property will not change in the presence of sufficient oxygen, a phenomenon known as the Warburg effect (3). Lactate has long been considered a metabolic waste product, but recent studies have identified lactate as one of the most significant metabolites in the tumor microenvironment that contributes to microenvironmental acidosis and immunosuppression. Malignant cell-produced lactic acid causes acidification of the tumor microenvironment (TME), promotes proliferation and accumulation of myeloid-derived suppressive cells (MDSCs), and inhibits the cytolytic function of effector cells (4). Lactic acid inhibits the differentiation and maturation of monocytes into dendritic cells, and several studies have confirmed the ability of lactic acid to induce polarization into M2-type macrophages (5–7). Lactic acid inhibits antigen presentation function by activating GPR81 in DC cells and inhibiting the production of cAMP, IL-6, IL-12, MHC-II, and other immunoreactive factors (8). A recent study reported that lactate inhibits RIG-I-like signaling and suppresses type I interferon production by inhibiting MAVS protein polymerization (9). Lactate signaling also promotes Treg differentiation and its mediated inhibition, promotes inflammatory Th17 cell differentiation, and inhibits the killing effect of CD8^+^ T cells and NK cells (10, 11). Moreover, lactate serves as an important carbon source for tumor cells as well as immune cells, and it is taken up by cells to mediate various intracellular signaling and function changes. Zhao et al. reported for the first time that lactate can also act as a modifying substrate to mediate lysine lactylation modification of histones under the action of histone acetyltransferase p300, which regulates the expression of genes related to macrophage polarization during immune activation (12, 13). Subsequently, HDAC1-3 was identified as the most potent lysine lactylation modification “eraser” (14).

The modern lifestyle has greatly changed the disease spectrum of cancer. In developed countries or urban areas, the incidence rate of cancer related to obesity and westernized lifestyle raised very high, including colorectal cancer, prostate cancer, kidney cancer and bladder cancer {Chen:fh}. Clear cell renal cell carcinoma (ccRCC) is a typical metabolic disorder tumor with robust lipid and glycogen accumulation, and recent studies have increasingly focused on the role of lactate-related metabolic factors in renal carcinogenesis. Lactate dehydrogenase, which catalyzes the production of lactate from pyruvate and is deeply involved in the regulation of the Warburg effect, was the first widely studied regulator in RCC. Hala et al. reported for the first time the correlation between LDHA upregulation and poor prognosis in RCC patients through tissue immunohistochemical studies (15). Zhao et al. discovered that LDHA was highly expressed in RCC tissues and that it mediated tumor metastasis by promoting epithelial-mesenchymal transition (EMT) (16, 17). Further research revealed that the promoting effect of lactate on EMT may be partially dependent on Sirtuin-1 activity inhibition (18). LDH reflects tumor tissue hypoxia and neovascularization levels and is widely used as a tumor load-related marker. A retrospective clinical trial reported that serum baseline LDH levels were an independent risk factor for postoperative PFS in patients with metastatic ccRCC treated with Nivolumab and were associated with poorer PFS in patients in the IMDC staging favorable group (19). Systematic reviews and meta-analyses announced that a high baseline serum LDH to lymphocyte ratio was independently correlated to the prognosis of metastatic RCC patients treated with tyrosine kinase inhibitors (TKI) (20, 21). Zhang et al. conducted a meta-analysis of lactate dehydrogenase’s prognostic role in metastatic RCC and found that high preoperative serum LDH levels were significantly correlated with poor postoperative overall survival (OS) and progression-free survival (PFS) (22). In addition, MCTs, which mediate the intercellular lactate shuttle, have also received extensive attention. By analyzing microarray data constructed from a large number of surgical specimens and performing immunohistochemical staining, Paul et al. demonstrated that MCT1 was an independent predictor of cancer-specific survival (CSS) in ccRCC (23). Overexpression of MCT1 and its partner CD147 have also been used to predict ccRCC progression (24). These findings suggest that lactate-related metabolism factors profoundly influence RCC progression and hold predictive value for frontline adjuvant therapy.

The value of lactate metabolism-related genes (LMRGs) in the prognosis of ccRCC has been recently investigated, but there are several shortcomings (25, 26). First, the lactate metabolism regulators discussed in prior publications were not comprehensive; second, their conclusions have not been validated in real-world patient cohorts treated with targeted therapy or immune checkpoint blockade (ICB); and, in addition, there is a lack of single-cell level investigation to elucidate the cell type specificity of lactate metabolism. In the present study, we explain the alteration of lactate metabolism-related processes and regulators with respect to different immune cell types. We proposed a novel prognostic index by constructing lactate-related gene pairs, which has exhibited robust stability and predictive power in previously published datasets because it does not depend on absolute gene expression level. Most importantly, validation in cohorts treated with TKI or ICB directly demonstrated the value of the lactate-related gene pair index (LRGPI) in guiding the selection of frontline adjuvant strategies.

## Materials and methods

### Data acquisition and pre-processing

In this study, we integrated several independent datasets for comprehensive analysis. We obtained transcriptomic and 450K methylation sequencing profiles and the Masked Copy Number segment file for the TCGA-KIRC cohort from the Xena portal as a development dataset. FPKM values were transformed into TPM values to maintain comparability with MicroArray platform-derived data. Gene expression and clinical profiles of E-MTAB-1980 and E-MTAB-3267 were obtained from the ArrayExpress portal (https://www.ebi.ac.uk/arrayexpress/) as the test set. In addition, several datasets were obtained from the GEO portal (https://www.ncbi.nlm.nih.gov/gds/?term=) for external validation. Specifically, six datasets based on the GPL570 platform (GSE36895, GSE53757, GSE66272, GSE73731, GSE46699, and GSE22541) were combined into the external validation set GPL570, and three datasets based on GPL10588 (GSE40435, GSE105261, and GSE65615) were merged into GPL10588. The “sva” function of the “Combat” package was used to merge data generated from the same platform to remove batch effects. The Checkmate cohort consisted of 181 cases of metastatic ccRCC treated with Nivolmab and 130 cases treated with Everolimus, with TPM transcriptome data and corresponding clinical information obtained from the report of Brau et al. (27). In addition, the phase III clinical trial Javelin-101 enrolled 726 cases of advanced RCC and compared the efficacy of Sunitinib and Avelumab plus Axitinib. The TPM transcriptome data and clinical outcome of Javelin-101 were obtained from Motzer et al. (28). The log ratio transformed proteomic expression data and their biospecimen information were downloaded from the CPTAC portal (https://proteomics.cancer.gov/programs/cptac) for protein level validation. Briefly, there were 83 normal samples and 111 ccRCC tumor samples in the CPTAC-ccRCC cohort. Representative normal kidney tissue and renal cancer pathology IHC slides were downloaded from the HPA portal (https://www.proteinatlas.org).

### Curation of genes involved in the lactate metabolism process

Six GO processes involved in lactate metabolism were archived and collected from MSigDB, including GO_LACTATION, GO_LACTATE_METABOLIC_PROCESS, GO_LACTATE_TRANSMEMBRANE_TRANSPORT, GO_LACTATE_TRANSMEMBRANE_ TRANSPORTER_ACTIVITY, GO_LACTATE_DEHYDROGENASE_ACTIVITY, GO_L_LACTATE_DEHYDROGENASE_ACTIVITY. In total, these gene sets contain 74 hub genes related to lactate metabolism.

### Differentially expressed genes analysis and genomic heterogeneity analysis

The “limma” package was used to perform DEG analysis on high throughout sequencing/Microarray-derived data. Nonsynonymous mutations of a single gene were extracted from the Mutect file and defined with reference to Brau et al (27). The methylation data were preprocessed with reference to the published report (29). The gene promoter region was defined as TSS1500, TSS200, 5’-UTR, and 1stExon. The median value of the promoter region probe was used to represent the promoter region methylation level of a single gene. The copy number variation (CNV) file and reference markers file were submitted to the GISTIC 2.0 modules in the GenePattern platform (https://cloud.genepattern.org/gp/pages/index.jsf) to perform CNV analysis. The Human Hg38 reference genome was set as a reference, the amplifications and deletions threshold were set at 0.3 (q value<0.05), and the confidence level was set at 0.99.

### Single-cell RNA sequencing data analysis

sc-RNA seq data for seven ccRCC tumors and six normal samples, along with cell type annotations, were stored as GSE159115 in the GEO database. The “Seurat” package was used to create single-cell objects for each sample, retaining cells with mitochondrial genes 25% of the time and nFeature_RNA > 300. The data of each sample was normalized, and 2000 genes with the highest variance were chosen based on variance stabilization transformation. Anchors were identified using the FindIntegrationAnchors function, and all samples were integrated into one Seurat object using the IntegrateData function to remove batch effects. The Seurat objects were then downscaled using the umap method, and visualization was done using the “scRNAtoolVis” package. Gene set enrichment assessment at the single-cell level was performed using the “AUCell” and “SCpubr” packages, respectively. Cell-specific molecular markers were identified by the FindAllMarkers function (logFCfilter=0.25, adjusted p-value <0.05, min.pct=0.25). CytoTRACE can assess the differentiation status of individual cells while also generating a numeric vector of each gene’s Pearson correlation with CytoTRACE (30). The degree of dedifferentiation of individual tumor tissues was assessed using a curated stem gene set of 109 genes with proliferation and immune-related genes removed as proposed by Miranda et al. (31).

### Western blotting

The human normal renal epithelial cell line HK2 and the tumor cell lines ACHN, 786-O, and OS-RC-2 were used for western blotting analysis in according to the standard procedure. The antibodies were purchased from the ABclonal Technology company (LDHA, #A0861, LDHB, #A7625).

### Lactate metabolism-related gene pairs construction

Gene alias in different datasets was manually checked to keep consistent with the current gene symbols in Genecard (https://www.genecards.org/), and we retained 64 lactate metabolism genes detected in all datasets to construct lactate-related gene pairs. The gene pair construction procedure was based on that previously reported in the literature, with some modifications to the details (32). The main steps include the following: 1) identification of genes significantly associated with OS using uni-variate Cox regression (p<0.05); 2) pairwise comparison of the prognostic gene Gi with all LMRGs Gj, and for each gene pair (Pij) starting with Gi, Scoreij=1 if Gi > Gj and 0 otherwise; 3) if Scoreij is consistent (i.e. Scoreij = 0 or 1) in more than 70% of the samples, then this gene pair was abolished. This method calculates scores based on the relative expression levels between gene pairs without considering absolute gene expression levels or data normalization methods, thus providing superior stability and cross-platform reproducibility.

### Development of a prognostic index using adaptive LASSO algorithm

**Figure 1:**
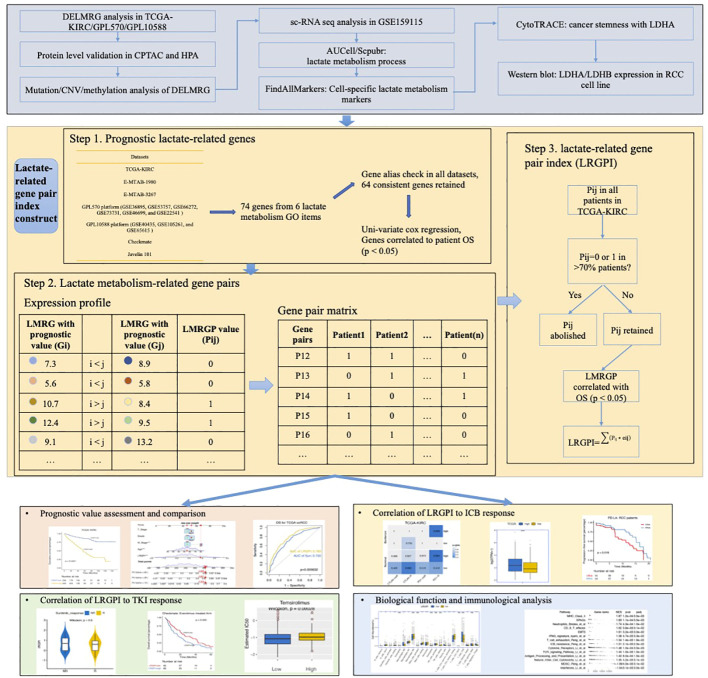
Graphical Abstract: The scheme of LRGPI construction and workflow of this study.

To enhance the stability of the prognostic index, patients with an OS time shorter than 1 month were excluded before the construction and validation of the prognostic index. LMRGPs found to be significantly associated with OS by performing univariate Cox regression (p < 0.05) were chosen as LRGPI candidates. To minimize the excessive penalty of parameters in traditional LASSO regression, we applied the adaptive lasso method to screen the best combination of parameters to construct LRGPI. Adaptive lasso is done using the “glmnet” package. Specifically, the process consists of the following steps: 1) run a 10-fold cross-validated ridge regression to obtain penalty weight coefficients; 2) run a 10-fold cross-validated LASSO regression based on the penalty weights. The adaptive lasso gives the optimal combination of parameters and the corresponding non-zero coefficients for reaching the minimum partial likelihood deviation, and LRGPI is calculated as the sum of LMRGP scores (1 or 0) multiplied by the corresponding coefficients. The scheme of LRGPI construction and workflow of this study is illustrated in **Figure 1**.

### Deconvolution of the immune infiltration and gene set activity evaluation

We used the “IOBR” package to analyze the TME components. IOBR integrated nine mostly used deconvolution methods, including CIBERSORT, EPIC, MCPcounter, xCELL, ESTIMATE, TIMER, quanTIseq, and IPS, and numeric published gene sets related to tumor metabolism, cancer hallmarks, TME, etc. (33). CIBERSORT was selected for deconvolution assessment of the level of infiltrating immune cells. The “ssgsva” algorithm built into this package was used for gene set enrichment to assess the activity of the tumor metabolism activity. Enrichment analysis of cancer hallmarks and TME was performed using the “fgsea” package.

### Predicting the correlation of LRGPI with frontline adjuvant therapy

The “pRRophetic” package was used to estimate the drug sensitivity of individual samples, as reported by Zhou et al. (34). Specifically, the transcriptomic data of urological cell lines and experimentally determined IC50 values for the target drugs were designated as standard data, and the package used the transcriptomic data of the samples to be tested to construct a ridge regression model, thereby deriving the estimated IC50 values of the samples to be tested. A gene expression pattern similarity comparison was performed using the Subclass Mapping module of the GenePattern portal. Specifically, 47 melanoma patients treated with CTLA4/PD-1 blockade and their drug response labels were designated as standards. Their transcriptome data and tags were submitted to the portal module together with the samples to be tested and grouping labels to obtain gene expression similarity test p-values (35).

### Statistical analysis

Visual image plotting and statistical analysis of this study were completed using R 4.1.1. The classic Kaplan-Meier curve was used to visualize patient survival status, while the log-rank test was used to differentiate survival differences between groups. The prognostic value of numerical or categorical factors was assessed using univariate or multivariate Cox regression models, and forest plots were drawn using the “forestplot” package. The nomogram under the univariate model was plotted using the “rms” package, and the predictive efficacy of the model was assessed using ROC curves and calibration curves. The heat map in the paper is drawn using the “ComplexHeatmap” package. We used the Wilcoxon test or the Kruskal-Wallis test for two-group or multi-group continuous variables, and a two-sided test with a p-value < 0.05 was considered a statistically significant difference. The Bonferroni correction was used to reduce the likelihood of Type I error in multiple replicate tests.

## Results

### Multi-omics analysis of differentially expressed lactate metabolism-related genes in ccRCC

**Figure 2:**
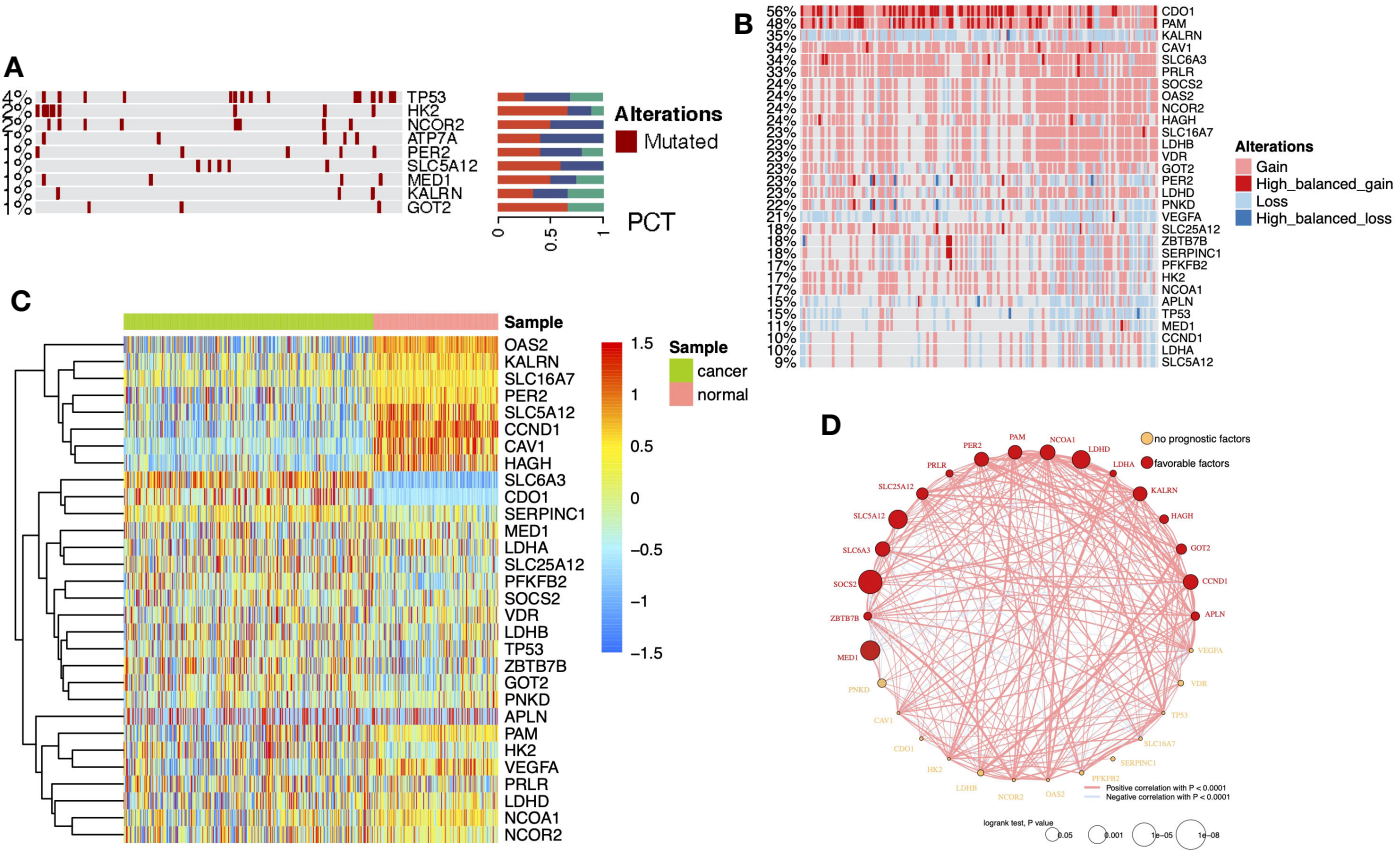
**(A)** Heatmap with bar plots displayed the mutation rates of DELMRGs. **(B)** Heatmap displayed the amplification and deletion landscape of 30 DELMGs. **(C)** Heatmap showed the promoter methylation level of 30 DELMGs. **(D)** Integrated network of correlation and prognostic value of the DELMRGs.

DEG analysis of the three cohorts (TCGA-KIRC, GPL570, and GPL10588) identified 30 DELMRGs (**Figures S1A–C**). The mutation landscape did not find high-frequency mutations in DELMRGs (**Figure 2A**, frequency > 5%), but CNV analysis revealed frequent gene copy number amplification and deletion (**Figure 2B**). Further, we examined the promoter methylation levels of DELMRGs and found that several genes (such as OAS2, KALRN, SLC16A7, PER2) had significantly lower methylation levels in tumor samples, while several genes (SLC6A3, CDO1, and SERPINC1) were significantly higher methylated (**Figure 2C**). Proteomic data verified that LDHA, HK2, NCOR2, CAV1, CCND1, OAS2, VEGFA, MED1, and PAM were significantly higher expressed, while PFKFB2, LDHB, PNKD, LDHD, HAGH, SLC16A7, SLC5A12, GOT2, and SLC25A12 were lower expressed in tumor samples (**Figure S1D**; **Supplementary Table 1**). In addition, the DELMRGs were further validated in protein expression level by their representative normal and tumor tissue staining slides in HPA portal (**Figure S2**). We then described the correlation and prognostic value of the DELMRGs using an integrated network (**Figure 2D**). Most DELMRGs interacted positively, and many genes, including MED1, ZBTB7B, SOCS2, SLC6A3, SLC5A12, SLC25A12, PRLR, PER2, PAM, NCOA1, LDHD, LDHA, KALRN, HAGH, GOT2, CCND1, and APLN played protective roles in patients’ overall survival (OS).

### Disordered lactate metabolism processes in different cell types

**Figure 3:**
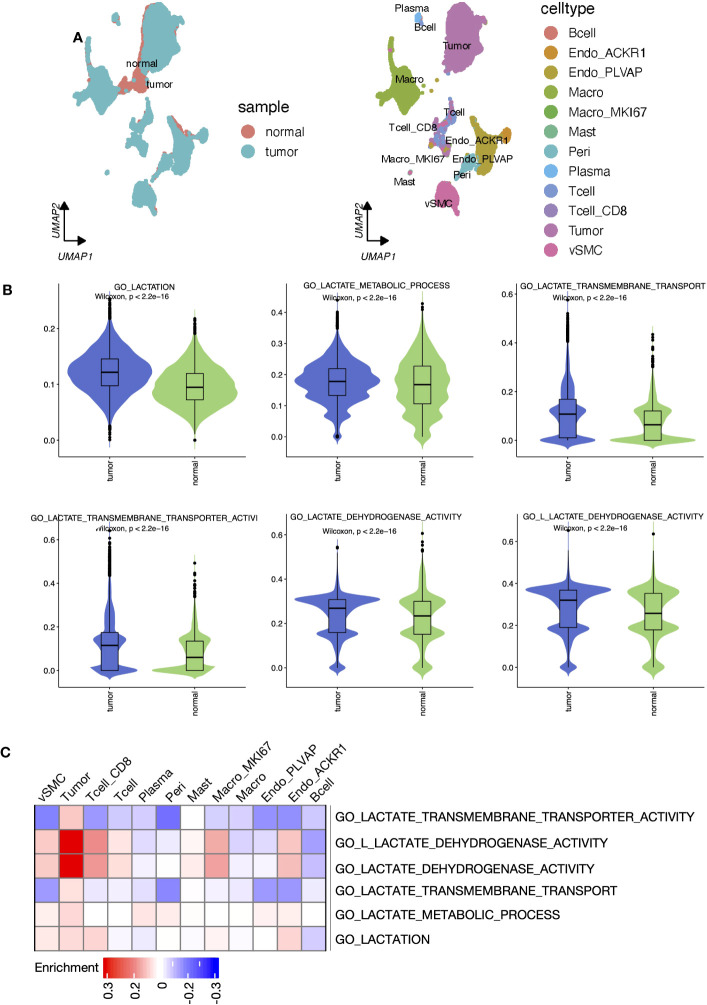
Lactate metabolism was activated in ccRCC tumor samples and different cell types. **(A)** UMAP reduction plots of cells grouped by sample and cell types. **(B)** The boxplots presented the “AUCell” scores that evaluated lactate metabolism processes at the single-cell level between tumor and normal samples. **(C)** Heatmap presented the enrichment scores produced by ‘Scpubr’ package in tumor sample-derived single cells.

Sc-RNA seq data can provide complementary information on cell-level variation beyond bulk-tissue sequencing data. According to the original literature (36), tumor tissue-derived cells were annotated into 13 cell types (**Figure 3A**). Each single cell was scored for six lactate metabolism-related biological process activities by the AUCell algorithm, and activities of these processes of the tumor tissue-derived cells were all significantly higher than those of the normal tissue-derived cells (**Figure 3B**). Further comparison across all tumor tissue cell types revealed that tumor cells, pericytes, endothelial cells, and vSMC cells hold the highest lactate metabolism activity than other cell types, while lactate transport across membranes was most active in tumor cells. The lactate dehydrogenase and L-lactate dehydrogenase activities of tumor cells, CD8^+^ T cells, vSMC cells, and MKI67^+^ macrophages were also higher than other cell types (**Figures S3A–F**). In addition, the enrichment results produced by “SCpubr” package highlighted the prominent lactate dehydrogenase and L-lactate dehydrogenase activities in tumor cells (**Figure 3C**).

**Figure 4:**
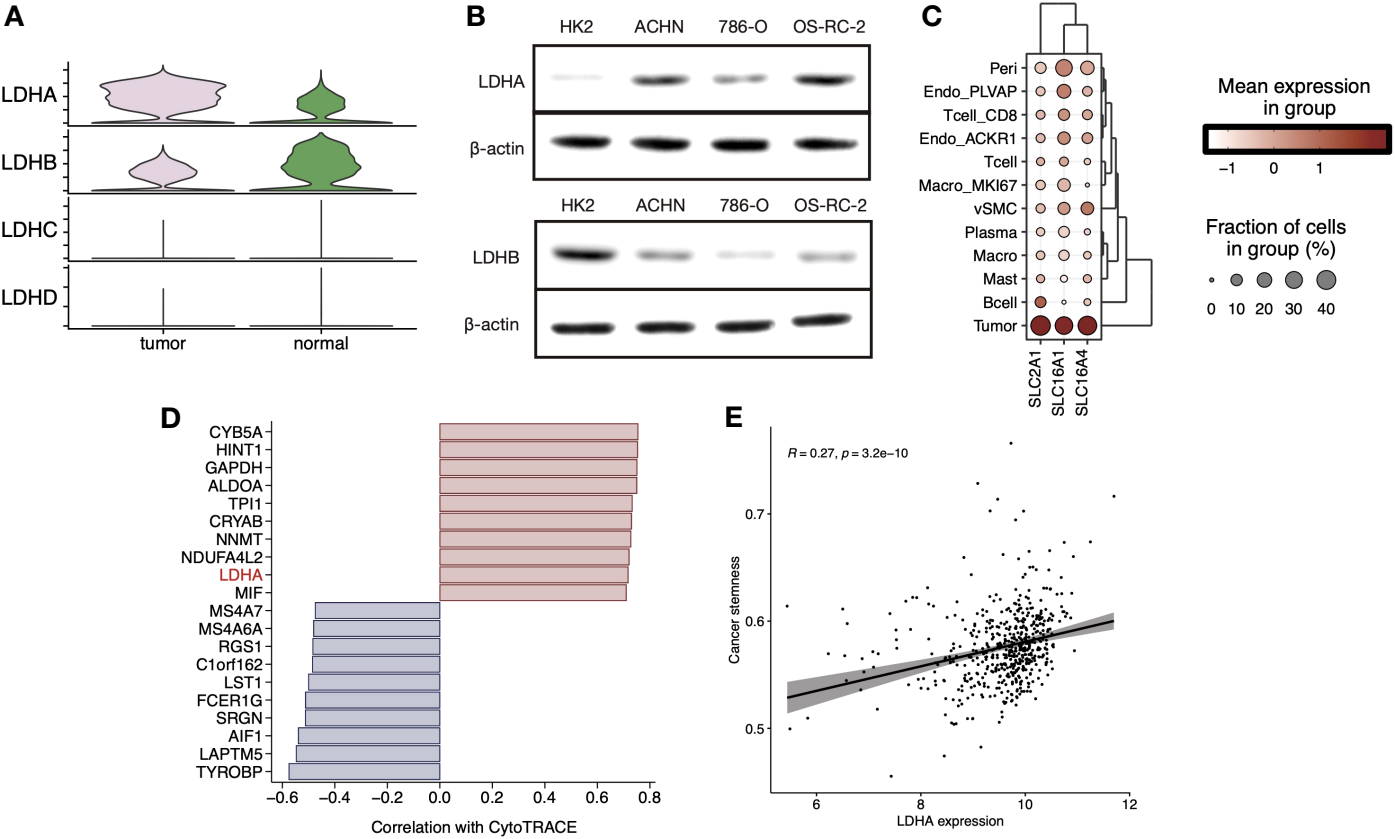
**(A)** Stacked violin plot displayed the expression level of LDHA, LDHB, LDHC, and LDHD in tumor and normal sample cells in sc-RNA Seq data (GSE159115), **(B)** LDHA and LDHB expression level were validated in RCC cell lines by Western blot. **(C)** Clustered dot plots of SLC2A1, SLC16A1, and SLC16A4 in tumor sample cells. **(D)** Bar plot showed the Pearson`s correlation of the top20 genes with dedifferentiation status calculated by CytoTRACE. **(E)** Scatter plot with a linear regression correlation of LDHA expression and cancer stemness evaluated by PNAS stem gene set in TCGA-KIRC.

We explored the expression levels of four major lactate dehydrogenase (LDHA, LDHB, LDHC, and LDHD) and found that LDHA was significantly higher expressed in tumor tissue-derived cells and LDHB was higher expressed in normal tissue-derived cells (**Figure 4A**). Further, cell line experiments showed a significant increase in LDHA expression and a significant decrease in LDHB expression in the tumor cell lines ACHN, 786-O, and OS-RC-2 relative to the human normal renal epithelial cell line HK2 (**Figure 4B**). Cell-specific molecular markers were then extracted (**Supplementary Table 2**). We observed the expression level of LMRGs in different cell types, and we found that LDHA was mainly expressed in tumor cells and ACKR1^+^ endothelial, while LDHB was highly expressed in vSMC, tumor cells, mast cells, and CD8^+^ T cells (**Figure S4**). Cells absorb glucose via GLUT1(SLC2A1), hypoxic cells release lactate through MCT4(SLC16A4), while tumor cells and endothelial cells absorb lactate through MCT1(SLC16A1). We explored the expression levels of transporter proteins in different cell types and found that GLUT1 and MCT1/4 were most highly expressed in ccRCC tumor cells (**Figure 4C**). These results suggest the idea that that lactate in the TME is mainly produced by vSMC and consumed and utilized by ccRCC tumor cells. Moreover, pericytes, endothelial cells, MKI67^+^ macrophages, and CD8^+^ T cells also hold a certain lactate uptake ability, suggesting the potential impact of lactate on their biological functions. In addition, the results of CytoTRACE showed that LDHA is highly correlated with inferred cancer stemness at the single-cell level (**Figure 4D**), and we verified the correlation at the bulk-tissue level using the TCGA cohort (**Figure 4E**).

### Establishment of a LRGPI to predict patients` prognosis

**Figure 5:**
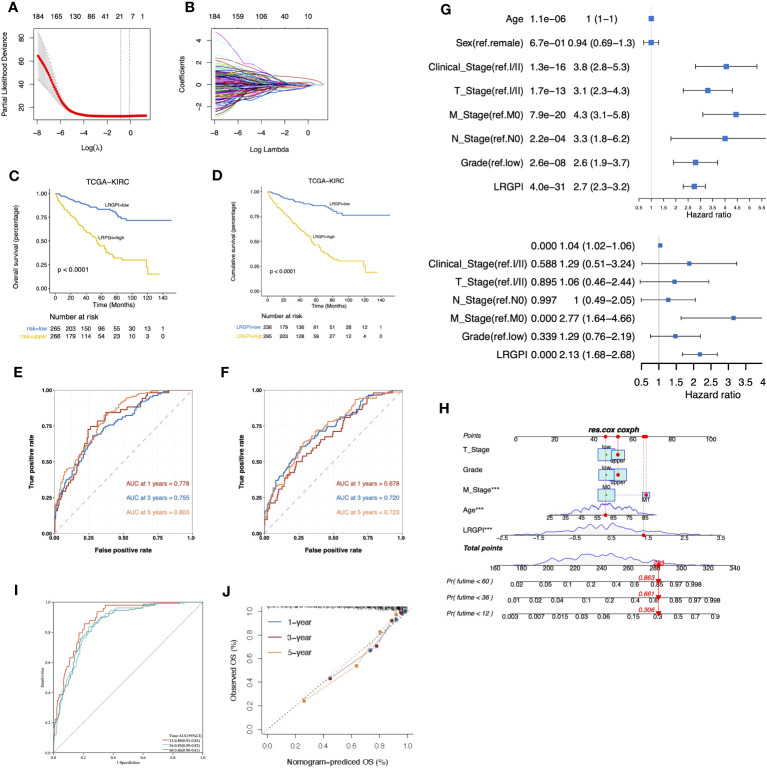
**(A, B)** The best combination of LMRG pairs was selected by adaptive-Lasso regression. **(C, D)** Survival analysis showed a different survival portion between the high- and low- LRGPI subgroups in TCGA-KIRC for OS **(C)** and DFS **(D)**. **(E, F)** Time-dependent ROC curves evaluated the prediction capacity of LRGPI for OS **(E)** and DFS **(F)** in TCGA-KIRC cohort. **(G)** Forest plots of uni- and multivariate Cox regression models demonstrated that LRGPI is an independent risk factor for patients` prognosis. **(H)** Nomogram to predict patients’ OS in TCGA-KIRC. The model incorporated the AJCC T stage, ISUP grade, metastatic status, patients’ age, and LRGPI. **(I)** Time-dependent ROC curves to evaluate the prediction capacity of the nomogram for patients` OS. **(J)** Calibration curves evaluated the prediction accuracy of the nomogram for patients` OS. ***p<0.001.

Here, we wonder whether the lactate metabolism-related prognostic index could be used to assess the clinical outcome of ccRCC patients. To minimize the impact caused by different sequencing platforms and standardization methods, we converted the lactate metabolism-related gene expression matrix into a gene pair matrix with values of 0 or 1. The univariate Cox regression identified 29 prognosis-related lactate metabolism genes. A total of 129 gene pairs were generated, and 96 gene pairs were significantly correlated to patients` OS. Adaptive lasso regression yielded the best combination of 18 gene pairs (**Figures 5A, B**, **Supplementary Table S3**). LRGPI was calculated as the method described. Patients were divided into high-and low-LRGPI groups based on the median LRGPI value, and the survival curves showed that patients in the high- LRGPI group had significantly lower overall survival (OS) and disease-free survival (DFS) rates than those in the low-LRGPI group (**Figures 5C, D**). The predictive efficiency of LRGPI for 1-year, 3-year, and 5-year prognosis reached 0.778, 0.755, and 0.803 for OS and 0.678, 0.720, and 0.723 for DFS, respectively (**Figures 5E, F**). LRGPI was proved to be an independent risk factor for patients` prognosis by adjusting clinical parameters in the multivariate Cox model (**Figure 5G**). To further improve the accuracy of clinical application, we integrated tumor T-stage, histologic grade, metastatic status, patient age, and LRGPI to construct a nomogram to predict patients` OS (**Figure 5H**). The ROC curve was used to assess the predictive capacity of the nomogram, and the area under the curve (AUC) reached 0.89, 0.85, and 0.86 for overall survival at 1, 3, and 5 years, respectively (**Figure 5I**). The predictive accuracy of the nomogram was further evaluated using the calibration curve, and the predicted OS status at 1, 3, and 5 years was found to be very close to the actual observation, indicating a robust predictive capability of the nomogram (**Figure 5J**).

**Figure 6:**
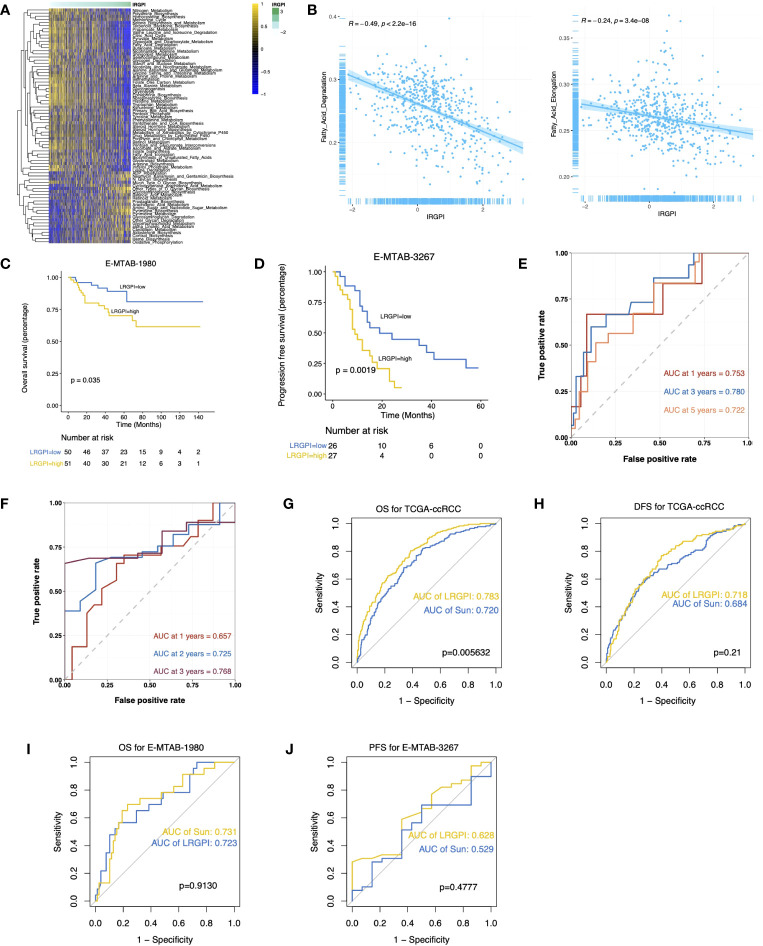
**(A)** Heatmap displays the differentially distributed cancer metabolism gene sets between high- and low-LRGPI subgroups in TCGA-KIRC cohort. The samples are ordered by LRGPI from lowest to highest. **(B)** Scatter plots with a linear regression correlation of LRGPI and fatty acid degradation and fatty acid elongation activity. **(C, D)** Survival analysis showed a different survival portion between the high- and low- LRGPI subgroups in E-MTAB-1980 for OS **(C)** and in E-MTAB-3267 for PFS **(D)**. **(E, F)** Time-dependent ROC curves evaluated the prediction capacity of LRGPI for OS in E-MTAB-1980 **(E)** and for PFS in E-MTAB-3267 **(F)**. **(G–J)** ROC curves displayed the prognosis prediction power of LRGPI and Sun`s lactate score for patients` clinical outcomes in several cohorts.

The GSEA results suggest activation of metabolic activities such as glycolysis, lipogenesis, fatty acid metabolism, and hypoxia in the high-LRGPI group samples, indicating high proliferation and energy demand in these samples. Meanwhile, immune response signals such as IFN responses, TGFβ signaling, IL2/STAT5 signaling, and KRAS signaling were also significantly upregulated (**Figure S5A**). To elucidate the association between LRGPI and cancer metabolism, enrichment scores for 103 tumor metabolism signals were calculated, and 75 signals were differentially distributed between the two groups (**Figure 6A**). We found that aerobic energy production pathways such as glycogen degradation, sugar degradation, the tricarboxylic acid cycle, pyruvate metabolism, and lactate degradation were significantly inhibited in patients with high LRGPI scores. At the same time, LRGPI was also negatively correlated to fatty acid degradation and long-chain fatty acid synthesis (**Figure 6B**), suggesting that fatty acids accumulated in the samples of the high-LRGPI subgroup.

### External validation and efficacy comparison of LRGPI

LRGPI were then generated for each tumor sample in E-MTAB1980 and E-MTAB3267 for external validation. Similar to the TCGA-KIRC, patients in the high-LRGPI subgroup identically showed significantly lower OS or PFS rates than patients in the low-LRGPI subgroup (**Figures 6C, D**). The predictive efficacy of LRGPI for 1-year, 3-year, and 5-year OS in E-MTAB-1980 was 0.753, 0.780, and 0.722, respectively (**Figure 6E**). For metastatic ccRCC patients treated by Sunitinib, the predictive power for PFS at 1, 2, and 3 years was 0.657, 0.725, and 0.768, respectively (**Figure 6F**). LRGPI remained an independent risk factor after adjusting clinical factors in E-MTAB1980 (**Figure S5B**). In addition, we applied the nomogram established in TCGA-KIRC to E-MTAB1980 (**Figure S5C**), and the calibration curve still observed a high degree of agreement between predicted and actual survival status (**Figure S5D**). The clinical nomogram achieved impressive predictive powers of 0.89, 0.92, and 0.87 for 1-year, 3-year, and 5-year OS, respectively (**Figure S5E**).

Sun et al. used 3 lactate metabolism genes (FBP1, HADH, and TYMP) to establish a prognostic signature to predict the ccRCC prognosis (26). We compared the predictive efficacy of Sun with LRGPI (**Figures 6G–J**). LRGPI achieved comparable or higher predictive power with Sun in all cohorts, but only in the TCGA-KIRC did the difference in the AUC value reach a statistically significant level.

### Correlation of LRGPI and targeted therapy for ccRCC patients

**Figure 7:**
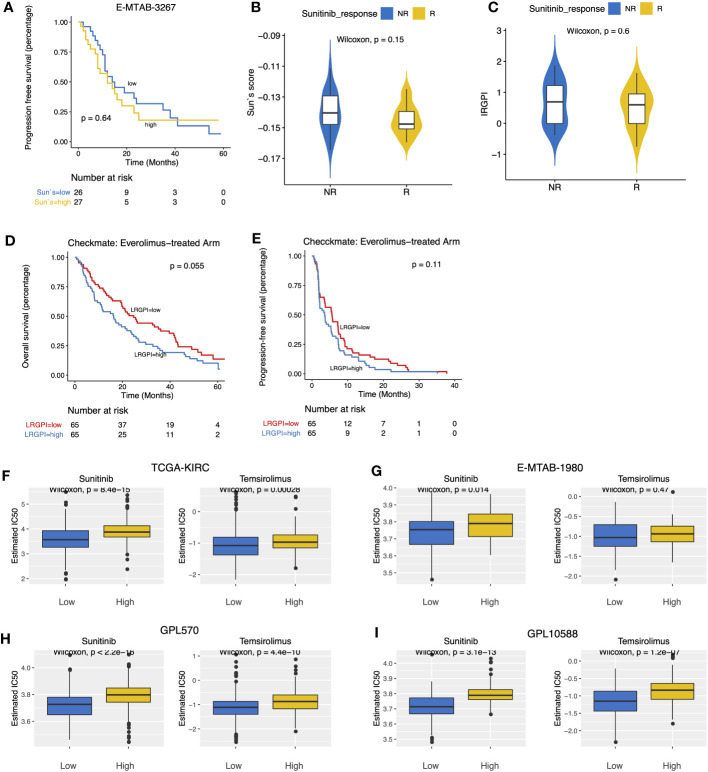
**(A)** Survival curves showed no significant difference in PFS between high- and low- Sun`s lactate score subgroups. **(B, C)** Boxplots displayed the comparison of Sun`s lactate score and LRGPI between the Sunitinib response and non-response samples. **(D, E)** Survival curves demonstrated that LRGPI was able to distinguish the OS **(D)** but not the PFS **(E)** in Everolimus-treated patients. **(F–I)** The predicted IC50 values of ccRCC samples for Sunitinib and Temsirolimus between high- and low- LRGPI groups in the TCGA-KIRC **(F)**, E-MTAB-1980 **(G)**, GPL570 **(H)**, GPL10588 **(I)** cohorts.

E-MTAB-3267 included 53 patients with metastatic ccRCC treated with sunitinib and responsive labels. For Sun`s lactate score, no significant difference in PFS between the high-and low-score groups was observed (**Figure 7A**). Patients were grouped according to Sunitinib responsiveness, and no significant differences in IRGPI or Sun`s lactate score were observed between the two groups (**Figures 7B, C**). The Checkmate trial documented clinical data from patients who failed initial treatment with sunitinib, and among 130 advanced ccRCC patients with complete records, we found that while no significant difference in PFS between the high-and low-IRGPI groups was observed, patients in the low-IRGPI subgroup showed extended OS time (**Figures 7D, E**). To further validate our findings, ridge regression was run to calculate the estimated IC50 values for each tumor sample in the 4 cohorts (TCGA, E-MTAB1980, GPL570, and GPL10558) using drug sensitivity data (IC50 values) provided by GDSC for urological tumor cell lines. Simulation results for the four cohorts consistently showed significantly lower IC50 values for Sunitinib and Temsirolimus in the low-IRGPI subgroup samples than in the high-IRGPI subgroup samples (**Figures 7F–I**). Validation results from drug-sensitivity simulation extrapolation and real-world cohorts exhibited a high degree of consistency in terms of mechanistic target of rapamycin (mTOR)-targeted agents, suggesting that patients in the low-IRGPI subgroup could benefit from mTOR-targeted therapy.

### Correlation of LRGPI and immunotherapy benefit for ccRCC patients

**Figure 8:**
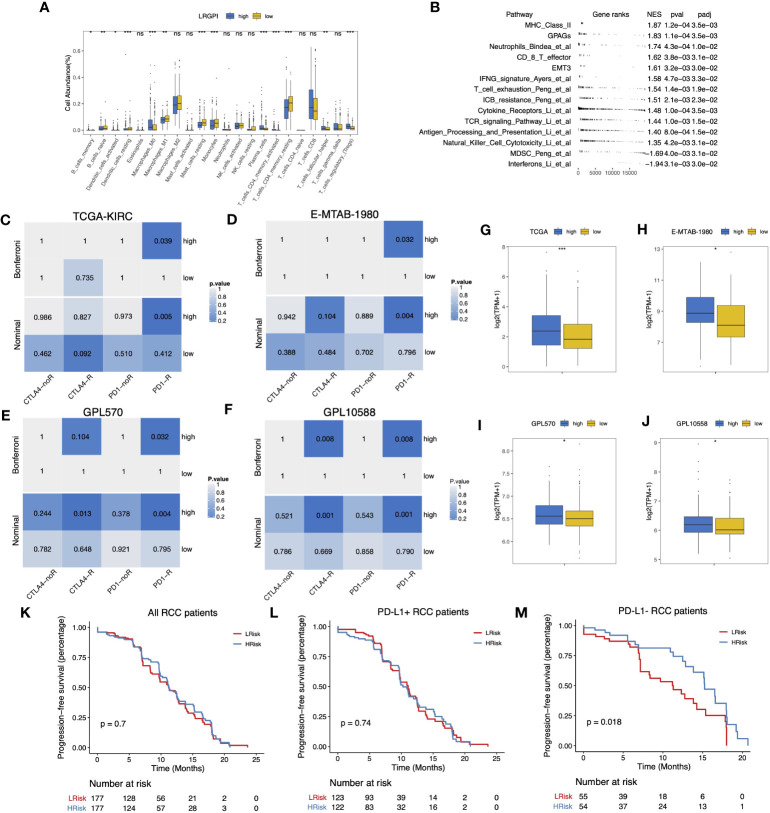
**(A)** Boxplot displayed the percentage of infiltrated immune cell types deconvoluted by CIBERSORT in TCGA-KIRC cohort. Wilcoxon test, ns, p>0.05, *p<0.05, **p < 0.01; ***p<0.001. **(B)** GSEA table of significantly altered TME-related gene sets between high- and low-LRGPI subgroups in TCGA-KIRC cohort. **(C–F)** Heatmaps displayed the nominal and Bonferroni-corrected p-values of Subclass mapping results in the TI-KIRC **(C)**, E-MTAB-1980 **I**, GPL570 **(E)**, GPL10588 **(F)** cohorts. **(G–J)** Boxplots showed that PD-1 was higher expressed in the high-LRGPI groups in the TCGA-KIRC **(G)**, E-MTAB-1980 **(H)**, GPL570 **(I)**, GPL10588 **(J)** cohorts. **(K–M)** Survival curves showed that LRGPI was able to distinguish the PFS in PD-L1- patients under Avelumab plus Axitinib treatment. ns, p<0.05.

According to the immunophenotype, inflammatory and lymphocyte-depleted ccRCC scored the lowest, and the wound-healing subtype scored the highest (**Figure S5F**). Using CIBERSORT to deconvolute the immune components, we found that the low-IRGPI subgroup harbored a significantly higher abundance of antigen-presenting cells (DCs, macrophages), monocytes, mast cells, and memory CD4^+^ T cell infiltrates, whereas the high-IRGPI subgroup had a higher abundance of Treg (**Figure 8A**). We then performed GSEA analysis of 119 TME-related signals and found significant activation of important immune activation markers such as angiogenic genes (GPAGs), effector CD8^+^ T, TCR signaling, antigen presentation signaling, MHC-II class signaling, natural killer cytotoxicity, and Merck18 signaling, as well as activation of ICB resistance signaling in the high-IRGPI subgroup (**Figure 8B**).

To clarify whether the immunoreactivity difference ultimately affects immunotherapy outcomes, we divided all patients in the Checkmate cohort into high- and low- LRGPI subgroups and found that patients in the low- IRGPI subgroup who received Nivolumab treatment showed no significant survival benefit beyond those who received Everolimus in terms of either PFS or OS (**Figures S6A, B**). However, patients in the high-IRGPI subgroup who received Nivolumab showed significantly longer OS time in comparison to those treated with Everolimus (**Figures S6C, D**). There are two possible explanations: either the high-IRGPI subgroup patients were not the best population to benefit from Everolimus, or the high-IRGPI subgroup patients had a significant response to Nivolumab. To test the above conjecture, we performed a gene expression profile comparison using melanoma samples treated with CTLA4/PD-1 blockade. The results showed that the gene expression profiles of the high-IRGPI samples showed significant concordance with PD-1-responsive melanoma samples in the four cohorts (**Figures 8C–F**, Bonferroni adjusted p-value < 0.05), demonstrating that the high-IRGPI samples were likely to respond to PD-1 blockade. High-IRGPI samples showed significantly higher levels of PD-1 expression in the four cohorts (**Figures 8G–J**).

The strategy of ICB plus TKI adjuvant therapy has pushed the adjuvant treatment of RCC into a new era, and the combination strategy is believed to be beneficial in reducing the multiple adverse effects of monotherapy and improving patient response rates (37). We then ask whether LRGPI has predictive value for the combination treatment outcomes. The Javelin-101 trial enrolled 354 RCC patients treated with Avelumab plus Axitinib. Patients` survival differences were not observed when grouping patients based on median LRGPI values (**Figure 8K**). When PD-L1 staining positive or negative subgroups were compared (**Figures 8L, M**), we were ecstatic to discover that the high-IRGPI subgroup had significantly longer PFS among PD-L1 patients. This is an interesting finding, and more prospective clinical trial cohorts are needed to further validate our findings in the future.

## Discussion

It has been well known that the lactate content of tumor tissue is higher than that of normal tissue, and lactate is necessary for cancer development. Lactate-related coding genes, or LncRNA, have been identified and shown to have predictive value in cancers such as colon cancer and lung cancer in thorough studies of tumor bulk tissue sequencing data (38, 39). The prognostic value of LMRGs in ccRCC was first discussed and reported by Sun et al. including 267 LMRGs involved in the lactate metabolic process, HP increased serum lactate, HP lactic acidosis, and HP lactic aciduria (26). Almost at the same time, Guo et al. focused on 27 genes involved in lactate metabolism and transport in ccRCC. In this presented report, we included 64 LMRGs from lactate metabolism, transporter proteins, and lactate dehydrogenase activity, which is a difference from previous reports (25). Here we constructed a novel prognostic index, the IRGPI, and demonstrated the advantage of the LRGPI in distinguishing the prognosis of ccRCC patients. Compared to the previously developed lactate-related scoring system, re-assigning gene pairs by comparing the relative expression levels of genes within each gene pair to construct a scoring system has stability across detection platforms, which means that individual tumor scores can be easily reproduced through quantitative RT-PCR in clinical practice (40).

The combined multi-omics analysis revealed that the different expression levels of these LMRGs can be partially attributed to changes in promoter methylation levels or to single-gene DNA copy number variation. We provide the first single-cell level evidence to confirm the disordered regulation of lactate metabolism, lactate transport processes, and lactate dehydrogenase activity in ccRCC tumor samples. We identified lactate metabolism markers for ccRCC cell types and found high expression levels of LDHA in tumor cells and CD8^+^ T cells. Besides tumor cells, MCT1 was also expressed on Pericytes and endothelial cells, while MCT4 was expressed on vSMC. Meanwhile, we observed highly active lactate dehydrogenase in tumor cells, ACKR1^+^ endothelial cells, MKI67^+^ macrophages, CD8^+^ T cells, and vSMC cells. Mild lactate metabolic activity was also observed in endothelial cells such as vSMC, Plasmacytes, Pericytes, and endothelial cells, but lactate transport was invariably inhibited in these cell types (**Figure 3C**). These findings suggest that vSMC cells act as the “producers” of lactic acid, while tumor cells and peripheral cells are the “consumers” under certain conditions. The phenomenon of tumor cells and vascular endothelial cells being able to survive, proliferate, and migrate in hypoxic environments has long been observed. The new vascular system can withstand the challenging environment of fluctuating oxygen tension because to the choice of respiratory-independent metabolism in endothelial cells. In addition, the accumulation of lactic acid in tumors is achieved by inhibiting PHD2 and activating HIF1-α and NF-κB, and to a large extent, it contributes to the angiogenesis phenotype (41). The driving force of lactic acid promoting angiogenesis provides new therapeutic options without the drawbacks of traditional anti-angiogenic drugs for ccRCC (42, 43). Interestingly, despite such high lactate dehydrogenase activity in T cells and MKI67^+^ macrophages, there is an apparent lack of lactate metabolic processes. Although this is consistent with the previously observed near absence of glycolytic activity in tumor-infiltrating T cells, no plausible mechanistic elaboration can be provided for this purpose (8, 44). Ubaldo et al. (45) first reported that lactic acid promotes stemness-related genes expression in breast cancer. Vineet et al. found that the LDHA product L-2 hydroxyglutamic acid (L-2HG) acts as an epigenetic modifier leading to H3 hypermethylation, thereby regulating stemness-related gene transcription in pancreatic tumors (46). To our knowledge, this is the first report to propose the correlation between LDHA and ccRCC stemness, and future elaborate experiments *in vivo* and *in vitro* are warranted to reveal the underlying mechanism.

Of note, Sun et al. reported that the low score samples were sensitive to Sunitinib and Temsirolimus, but did not validate their inference in a real-world cohort. We replicated the Sun`s lactate score and validated it in E-MTAB-3267, but unfortunately failed to find the efficacy of the Sun`s score in distinguishing sunitinib responsiveness or patients’ prognosis. IRGPI was sufficient to distinguish the prognosis of Sunitinib-treated patients, but the difference between responders and non-responders in the real-world cohort also did not reach statistical significance. One possible reason is that the number of cases included in E-MTAB-3267 was too small, and further validation in a larger cohort is desired. Our drug sensitivity simulation inference and real-world cohort validation confirmed the responsiveness of the low-LRGPI samples to Temsirolimus. The PI3K-Akt-mTOR-HIF axis drives cellular glycolysis and the Warburg effect, and its dysregulation is common in carcinogenesis (47). Thus, the intrinsic link between LRGPI and the responsiveness of mTOR-targeted therapy is not difficult to understand.

Proliferating tumor cells and activated immune cells exhibit enhanced metabolic activity, taking up large amounts of glucose to generate lactic acid via the Warburg effect and transporting the products for uptake by surrounding cells as energy-consuming substances or anabolic substrates. The idea that harmful lactate accumulation in the TME is one of the main causes of immunosuppression has been widely recognized (47, 48). Lactate accumulation inhibits the viability and cytotoxic products of antitumor effector cells, such as CD8^+^ T cells and NK cells, and promotes the differentiation and expansion of immunosuppressive cell populations, such as Treg, TAM, and MDSC (47). We investigated the relationship between LRGPI and the TME of ccRCC and found decreased antigen-presenting cells (DC, M1) and increased Treg/Tfh infiltration, as well as activation of CD8^+^ T effector signaling, TCR signaling, IFN signaling, antigen-presenting signaling, and NK cytotoxicity in high-LRGPI samples. We also observed elevated PD-1 expression in high-IRGPI samples and similar gene expression patterns to PD-1-blockade responders. The inference and external validation demonstrate that high-LRGPI ccRCC samples would benefit from PD-1 blockade therapy. Recently, Kumagai et al. reported that lactate upregulates PD-1 expression on Treg through massive uptake by MCT1 but inhibits PD-1 expression in CD8^+^ T cells. PD-1 blockade leading to Treg activation but not CD8^+^ T cells is the main mechanism of lactate-induced ICB failure (49). In addition, it has been demonstrated that ICB plus lactate blockade/lactate dehydrogenase inhibitors synergistically reduce Treg function, resulting in a more potent anti-tumor capacity than ICB monotherapy (11). The use of the LDH inhibitor oxamate to reduce lactate production in combination with pembrolizumab significantly increased CD8^+^ T cell infiltration in a humanized mouse model of non-small cell lung cancer and enhanced the effect of pembrolizumab monotherapy (50). We are aware that there are still no animal studies or clinical trials to test the lactate-targeted regimen in RCC. Based on these pan-cancer study facts, it is interesting to introduce the lactate-targeted strategy to RCC. Notably, lactate and its products have significant cell type-specific effects (51, 52). More detailed preclinical experiments are urgently needed to explore the biological response of immune cells to lactic acid changes before designing intervention strategies to precisely manipulate anti-tumor immunity. We have examined the guiding utility of LRGPI for patients undergoing combination therapy with ICB + TKI for the first time, in contrast to earlier studies on the establishment of prognostic markers for ccRCC. Interestingly, the findings showed that LRGPI is a significant factor in determining how patients with PD-L1-RCC would fare. Patients with greater oxygen transport and lipid metabolism activity in the arm receiving the combination of avelumab and Axitinib had longer PFS, according to the original study by Mozter et al (28). In an era where personalized treatment is increasingly emphasized, patient subgroup analysis reveals that the patient population for which the scoring system is applicable has more positive clinical significance.

This study has the inherent shortcomings of retrospective bioinformatics studies; using real-world tumor samples for PCR quantification of the LGRPI proposed in this study and validation of its prognostic guiding value in ccRCC, or even RCC of different pathological types, in different therapeutic contexts, will be the focus of the next research efforts. In immune cells, the lactate signaling pathway may be the link between metabolism and immunity. For example, how are key lactate metabolism and transport molecules such as LDGA, GLUT1, MCT1, and MCT4 expressed in renal cancer cells, vSMC, endothelial cells, macrophages, CD8^+^ T cells, and what are the effects on lactate uptake and utilization in cells? How does lactate in the microenvironment affect the aggregation and functional activation of immune cell populations dominated by macrophages and CD8^+^ T cells? Due to article length constraints, exploration of the specific mechanisms by which lactate promotes kidney cancer progression and modulates the immune microenvironment will also be the focus of the next study.

## Conclusion

Altogether, this study illustrates the cellular specificity of lactate metabolism in ccRCC at the single-cell level. We proposed that LRGPI could be used to not only predict prognosis but also effectively distinguish frontline therapy responders in ccRCC patients.

## Data availability statement

The datasets presented in this study can be found in online repositories. The names of the repository/repositories and accession number(s) can be found in the article/**Supplementary Material**.

## Author contributions

XL: Conceptualization, Formal Analysis, Investigation, Supervision, Writing – review & editing. GD: Data curation, Formal Analysis, Investigation, Methodology, Writing – original draft. LL: Validation, Visualization, Writing – review & editing. KP: Conceptualization, Data curation, Formal Analysis, Supervision, Writing – original draft.

## Supplementary material

Supplementary Figure 1
**(A-D)** Volcano plots of DELMRGs identified in the TCGA-KIRC, GPL570, GPL10588, and CPTAC-KIRC datasets. For CPTAC-KIRC cohort **(D**), protein expression level of PER2, SLC6A3, CDO1, SOCS2, TP53, APLN, and PRLR were not detected. Up-/down-regulated and not significantly altered LMRGs were labeled in red, blue, and yellow, respectively.

Supplementary Figure 2 Representative normal and tumor tissue slides in HPA portal demonstrated the overall staining level of LDHA, HK2, NCOR2, CAV1, CCND1, OAS2, VEGFA, MED1, and PAM were significantly higher, while PFKFB2, LDHB, PNKD, LDHD, HAGH, SLC16A7, SLC5A12, GOT2, and SLC25A12 were lower in tumor renal cancer samples than normal samples.

Supplementary Figure 3
**| (A-F)** Violin plots of the AUCell-evaluated gene set enrichment scores in cell types of tumor samples.

Supplementary Figure 4 Cell-specific molecular markers were identified by the FindAllMarkers function. Stacked violin plot displayed the expression level of key lactate metabolism-related genes in different cell types.

Supplementary Figure 5
**(A)** GSEA table of significantly altered cancer hallmarks between high- and low-LRGPI subgroups in TCGA-KIRC cohort. **(B)** Forest plots of uni-and multi-variate Cox regression models demonstrated that LRGPI is an independent risk factor for patients` OS in E-MTAB-1980. **(C)** Nomogram to predict patients’ OS in E-MTAB-1980. The model incorporated the AJCC T stage, ISUP grade, metastatic status, patients’ age, and LRGPI. **(D)** Calibration curves evaluated the prediction accuracy of the nomogram for patients` OS in E-MTAB-1980. **(E)** Time-dependent ROC curves evaluated the prediction capacity of the nomogram for patients` OS in E-MTAB-1980. **(F)** Boxplot displayed the LRGPI of predefined immune subtypes of ccRCC.

Supplementary Figure 6
**(A, B)** No significant survival difference was observed between Nivolumab- and Everolimus-treated groups in low-LRGPI subgroup patients. **(C, D)** Nivolumab showed significant overall survival benefit over Everolimus in high-LRGPI subgroup patients.

Supplementary Table 1 The differentially expressed proteins identified in CPTAC-KIRC cohort.

Supplementary Table 2 Cell markers of different cell types identified in sc-RNA seq GSE159115.

Supplementary Table 3 The gene pairs and coefficients to replicate the LRGPI.

## Glossary

**Table:**

CPTAC	Clinical Proteomic Tumor Analysis Consortium
HPA	the Human Protein Atlas
MDSC	myeloid-derived suppressive cell
TAM	tumor-associated macrophage
TME	tumor microenvironment
EMT	epithelial-mesenchymal transition
ccRCC	Clear cell renal cell carcinoma
OS	overall survival
PFS	progression free survival
CSS	cancer-specific survival
DFS	disease-free survival
LMRG	lactate metabolism-related genes
ICB	Immune checkpoint blockade
LMRGP	lactate metabolism-related gene pair
LRGPI	Lactate-related gene pair index
Sc-RNA	seg Single-cell RNA sequencing
FPKM	fragments Per Kilobase Million
TPM	transcripts Per Kilobase Million
CNV	copy number variation
DEG	differentially expressed gene
DELMRG	differentially expressed lactate metabolism-related gene
AUC	area under curve
ROC	receptor operative curve
ssGSEA	Single sample gene set enrichment analysis
TKI	Tyrosine kinase inhibitors
mTOR	Mechanistic target of rapamycin
