# Supplementary figures and images for: Cellular specificity of lactate metabolism and a novel lactate-related gene pair index for frontline treatment in clear cell renal cell carcinoma
Source: Front Oncol. 2023 Sep 19;13:1253783. doi: 10.3389/fonc.2023.1253783 (PMC10546032; doi:10.3389/fonc.2023.1253783)

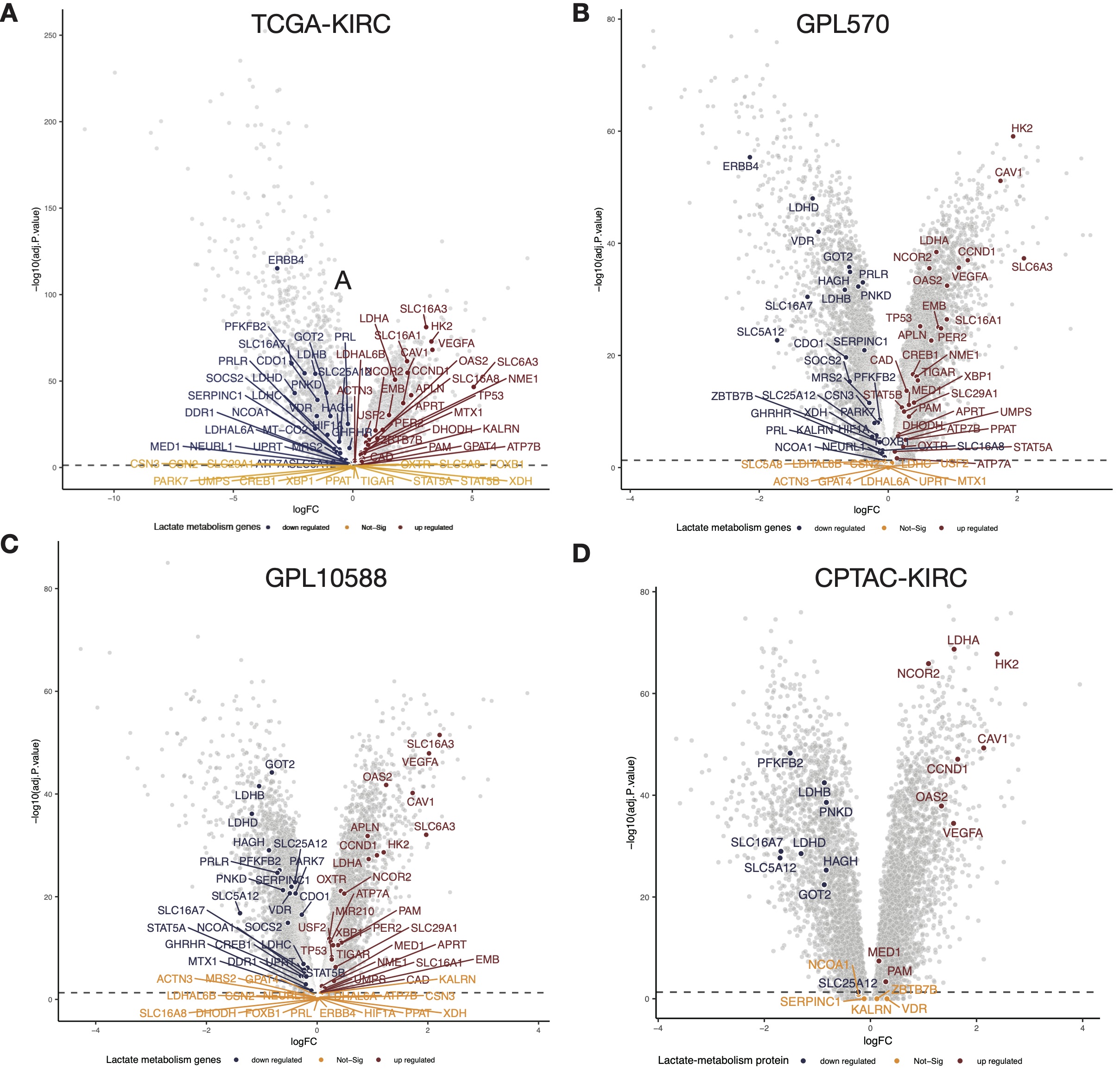

Supplement: Supplementary Figure 1 — (A-D) Volcano plots of DELMRGs identified in the TCGA-KIRC, GPL570, GPL10588, and CPTAC-KIRC datasets. For CPTAC-KIRC cohort (D), protein expression level of PER2, SLC6A3, CDO1, SOCS2, TP53, APLN, and PRLR were not detected. Up-/down-regulated and not significantly altered LMRGs were labeled in red, blue, and yellow, respectively. [file Image_1.jpeg]

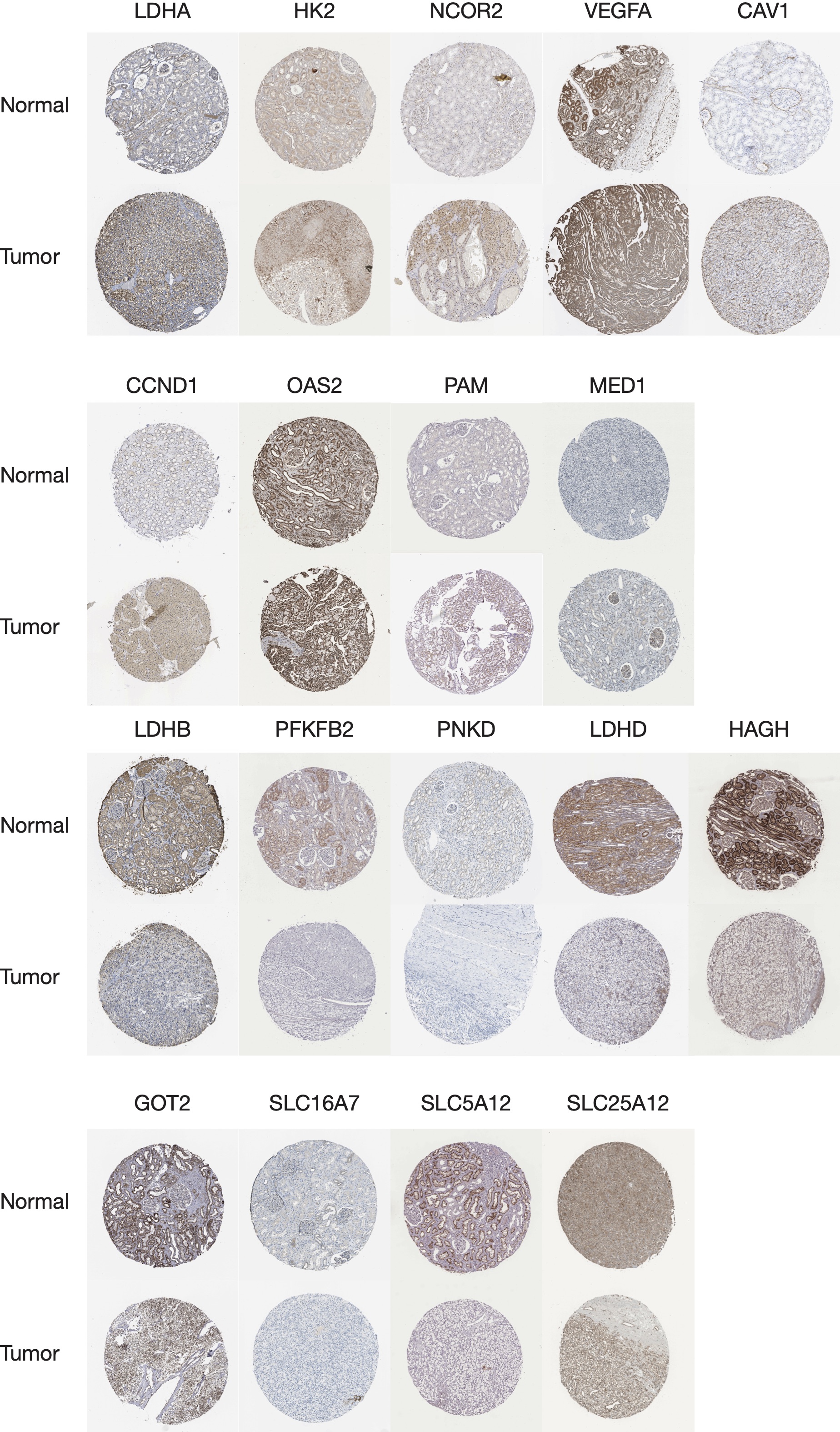

Supplement: Supplementary Figure 2 — Representative normal and tumor tissue slides in HPA portal demonstrated the overall staining level of LDHA, HK2, NCOR2, CAV1, CCND1, OAS2, VEGFA, MED1, and PAM were significantly higher, while PFKFB2, LDHB, PNKD, LDHD, HAGH, SLC16A7, SLC5A12, GOT2, and SLC25A12 were lower in tumor renal cancer samples than normal samples. [file Image_2.jpeg]

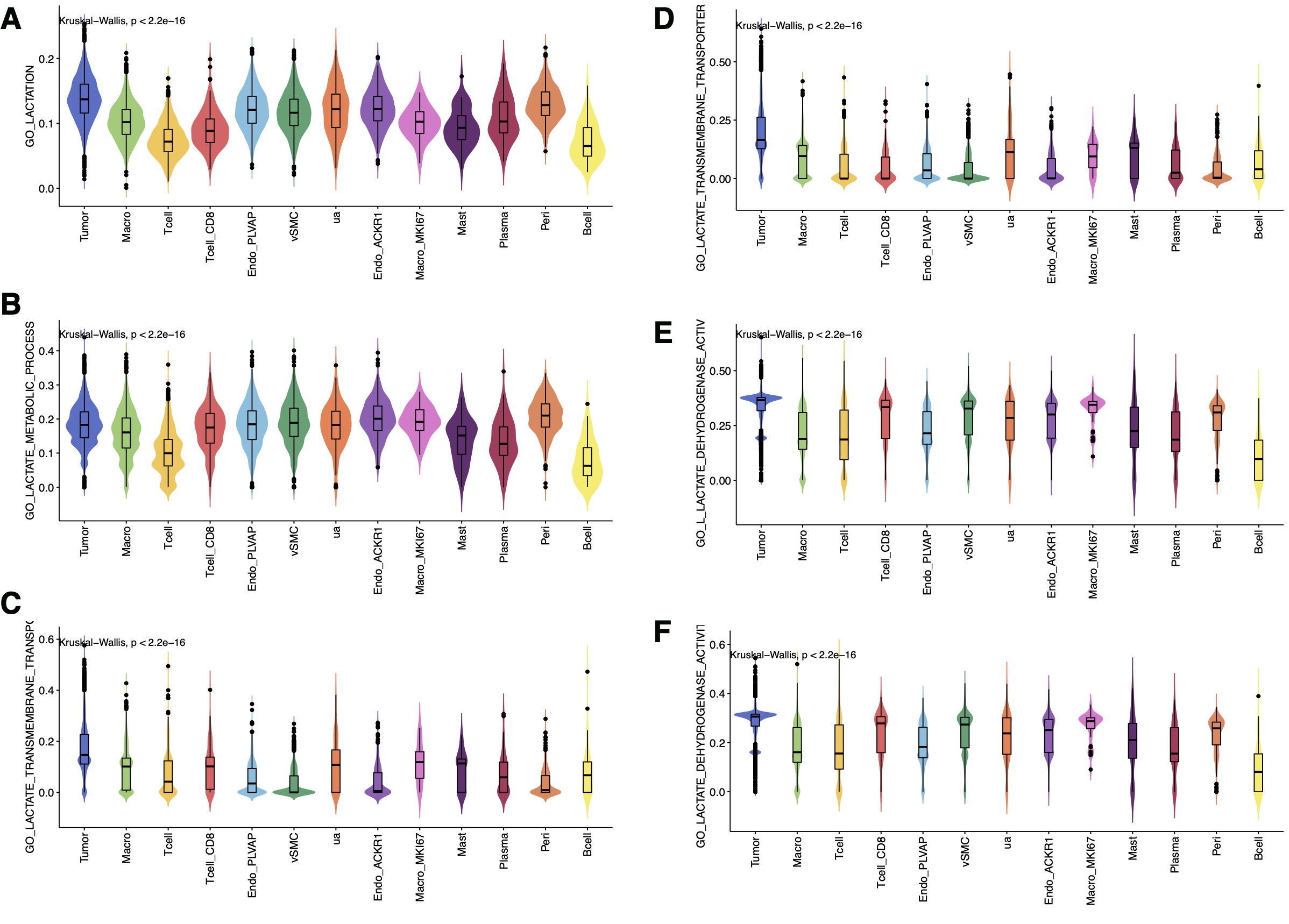

Supplement: Supplementary Figure 3 — | (A-F) Violin plots of the AUCell-evaluated gene set enrichment scores in cell types of tumor samples. [file Image_3.jpeg]

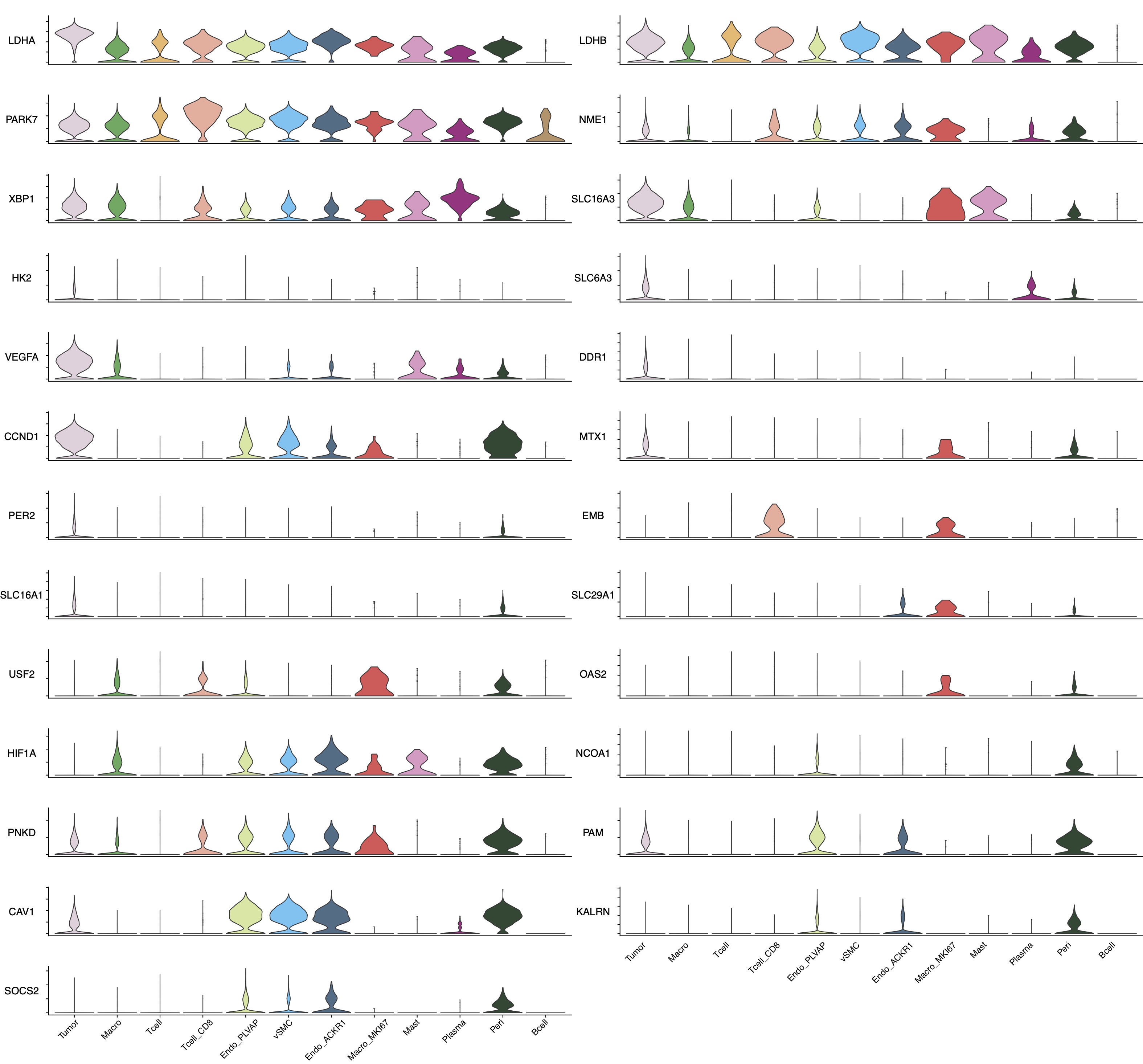

Supplement: Supplementary Figure 4 — Cell-specific molecular markers were identified by the FindAllMarkers function. Stacked violin plot displayed the expression level of key lactate metabolism-related genes in different cell types. [file Image_4.jpeg]

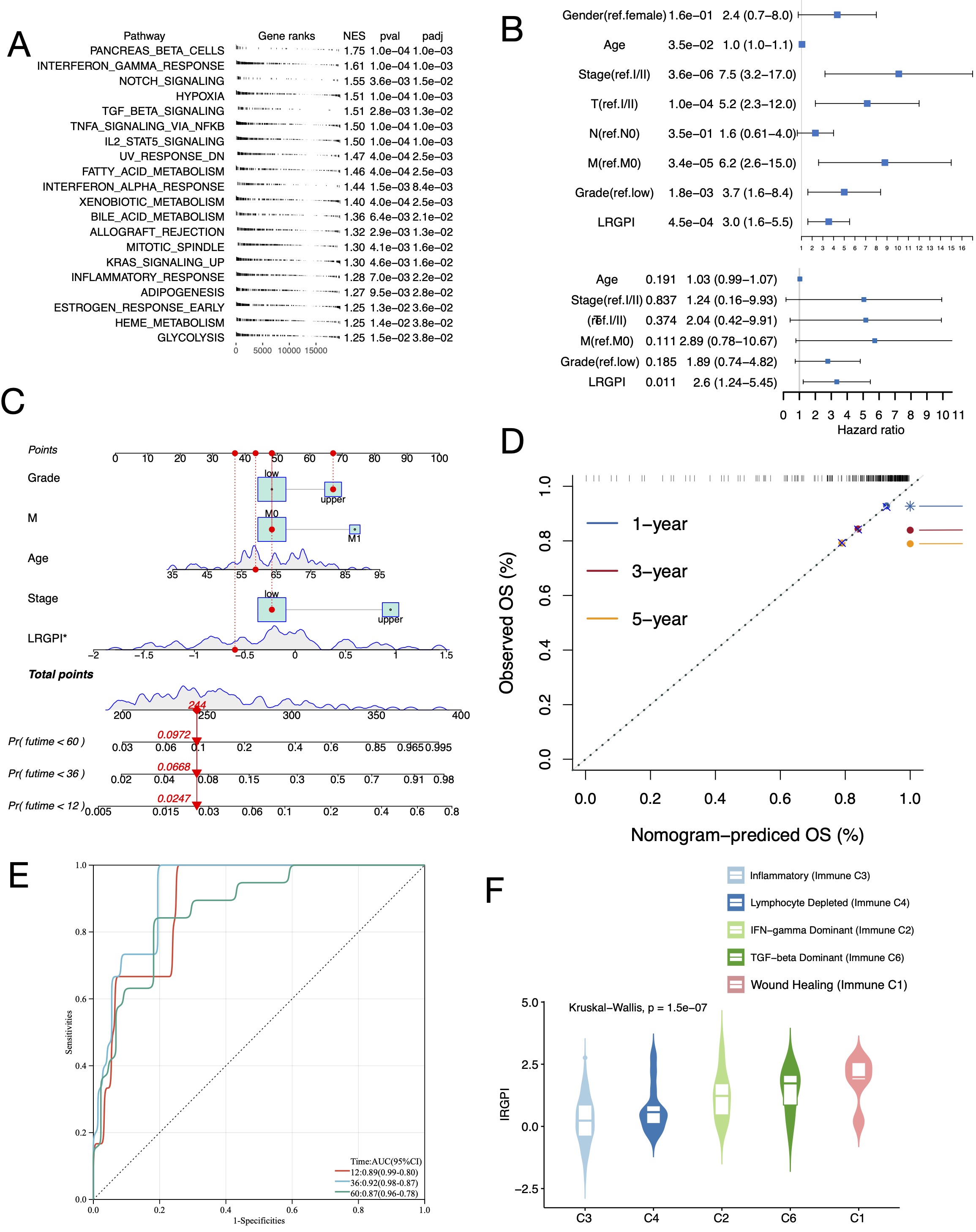

Supplement: Supplementary Figure 5 — (A) GSEA table of significantly altered cancer hallmarks between high- and low-LRGPI subgroups in TCGA-KIRC cohort. (B) Forest plots of uni-and multi-variate Cox regression models demonstrated that LRGPI is an independent risk factor for patients` OS in E-MTAB-1980. (C) Nomogram to predict patients’ OS in E-MTAB-1980. The model incorporated the AJCC T stage, ISUP grade, metastatic status, patients’ age, and LRGPI. (D) Calibration curves evaluated the prediction accuracy of the nomogram for patients` OS in E-MTAB-1980. (E) Time-dependent ROC curves evaluated the prediction capacity of the nomogram for patients` OS in E-MTAB-1980. (F) Boxplot displayed the LRGPI of predefined immune subtypes of ccRCC. [file Image_5.jpeg]

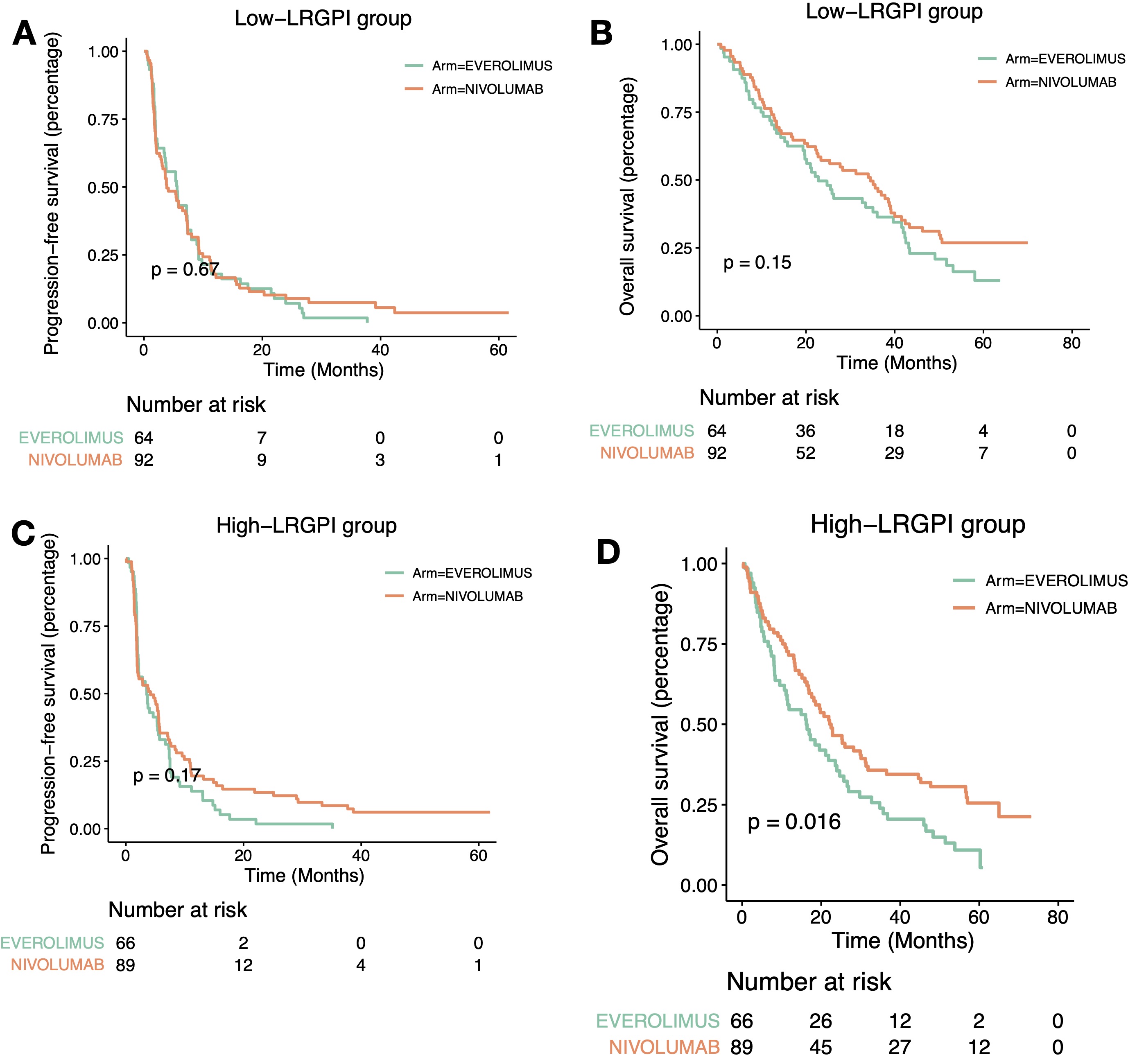

Supplement: Supplementary Figure 6 — (A, B) No significant survival difference was observed between Nivolumab- and Everolimus-treated groups in low-LRGPI subgroup patients. (C, D) Nivolumab showed significant overall survival benefit over Everolimus in high-LRGPI subgroup patients. [file Image_6.jpeg]
